# Supplementary figures and images for: Detecting and distinguishing between apicultural plants using UAV multispectral imaging
Source: PeerJ. 2023 Apr 14;11:e15065. doi: 10.7717/peerj.15065 (PMC10108856; doi:10.7717/peerj.15065)

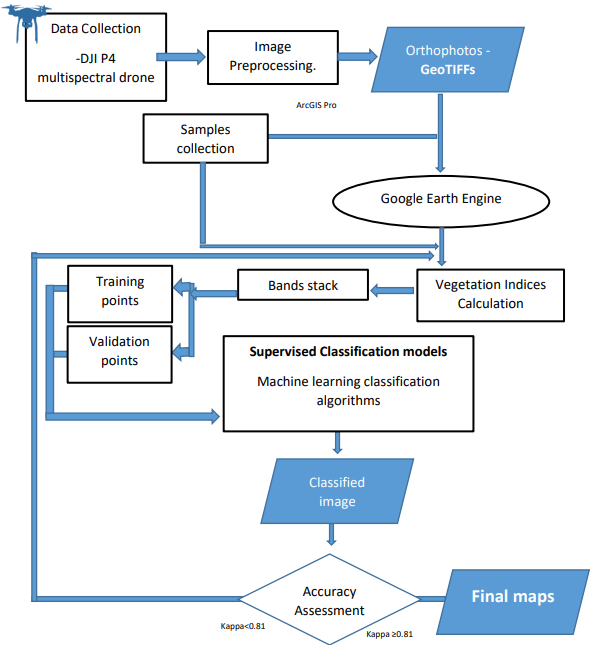

Supplement: Supplemental Information 1 [file peerj-11-15065-s001.png]
